# Delayed Shift in Microbiota Composition in a Marine Microcosm Pollution Experiment

**DOI:** 10.1007/s00284-024-03869-5

**Published:** 2024-09-18

**Authors:** Luis Yndy Ariem Ramirez, Inga Leena Angell, Tonje Nilsen, Knut Rudi

**Affiliations:** Faculty of Chemistry, Biotechnology and Food Science, University of Life Sciences, Ås, Norway

## Abstract

Benthic habitats are the largest habitats on Earth, being essential for marine ecosystem functioning. Benthic habitats are particularly vulnerable towards pollution and anthropogenetic influence due to general oligotrophic nature. We, therefore, simulated pollution events involving nitrate and sulphate, in combination with organic carbon. We then observed the microbiota composition the following month. Surprisingly, upon nitrate addition, an abrupt response was observed between two and three weeks after the pollution event. We observed a threefold reduction in species richness, with a dominance of the genus *Pseudarchobacter* within the Campylobacteriota phylum, concurring with a decrease in nitrification potential and an increase in Dissimilatory Nitrate Reduction to Ammonium (DNRA) and a regain in denitrification. Likewise, addition of sulphate contributed to a delayed response with reduction in species richness albeit weaker than for nitrate, leading to a shift towards potential spore-forming Firmicutes. There was also an increase in DNRA, but only for the oxic conditions, concurring with a regain in sulphate reductio and denitrification. For the nitrate addition experiments, the delay in response could potentially be attributed to the genus *Pseudarchobacter* which rely on sulphides for denitrification, while for the sulphate addition experiments, the delayed response might be explained by the germination of spores. The late increase of DNRA may indicate a shift towards a different metabolic regime for nitrogen. In conclusion, our microcosm experiments revealed delayed abrupt microbiota shifts resembling tipping points that can potentially be overlooked in natural ecosystems.

**Supplementary Information:**

The online version contains supplementary material available at 10.1007/s00284-024-03869-5.

## Introduction

Benthic ecosystems represent the boundary between the seafloor and the water column, being important for marine ecosystem functioning [16]. These habitats are the largest habitats on Earth, and they are particularly vulnerable to pollution and anthropogenic influence [2]. Human activities have a significant negative impact on the marine environment, particularly through pollution by organic waste [34]. Despite the widespread recognition of the impact of organic waste, our comprehension of the response and resilience of the marine microbiota is limited [19]. Especially important is the identification of whether the reaction to environmental pollution occurs gradually or suddenly [14], along with the time elapsed between a pollution event and its subsequent response.

Recent research suggests that marine ecosystems exhibit bistability, characterized by two distinct stable states governed by different sets of organisms and metabolic pathways. The environmental drivers of this bistability, however, are not well defined (van [31]). A major reason for this knowledge gap is the difficulty in analyzing abrupt changes from an ecological state to another [24]. Additionally, in many cases, it may be impossible to determine the precise impact of individual factors in natural systems [10]. Therefore, microcosm are experiments needed to decipher factors and responses connected to marine pollution [17].

The aim of this paper is to study abrupt pollution events, perturbating the benthic microbiota in marine ecosystems. This was done by adding both organic and inorganic nutrients in a microcosm experiment to simulate the main biogeochemical processes in the sea, including redox reactions of nitrogen and sulphur [25, 33]. Since oxygen is a key factor for the biogeochemical reactions [5], our investigations were conducted in both oxygen-rich (oxic) and oxygen-depleted (anoxic) environments.

**Fig. 1:**
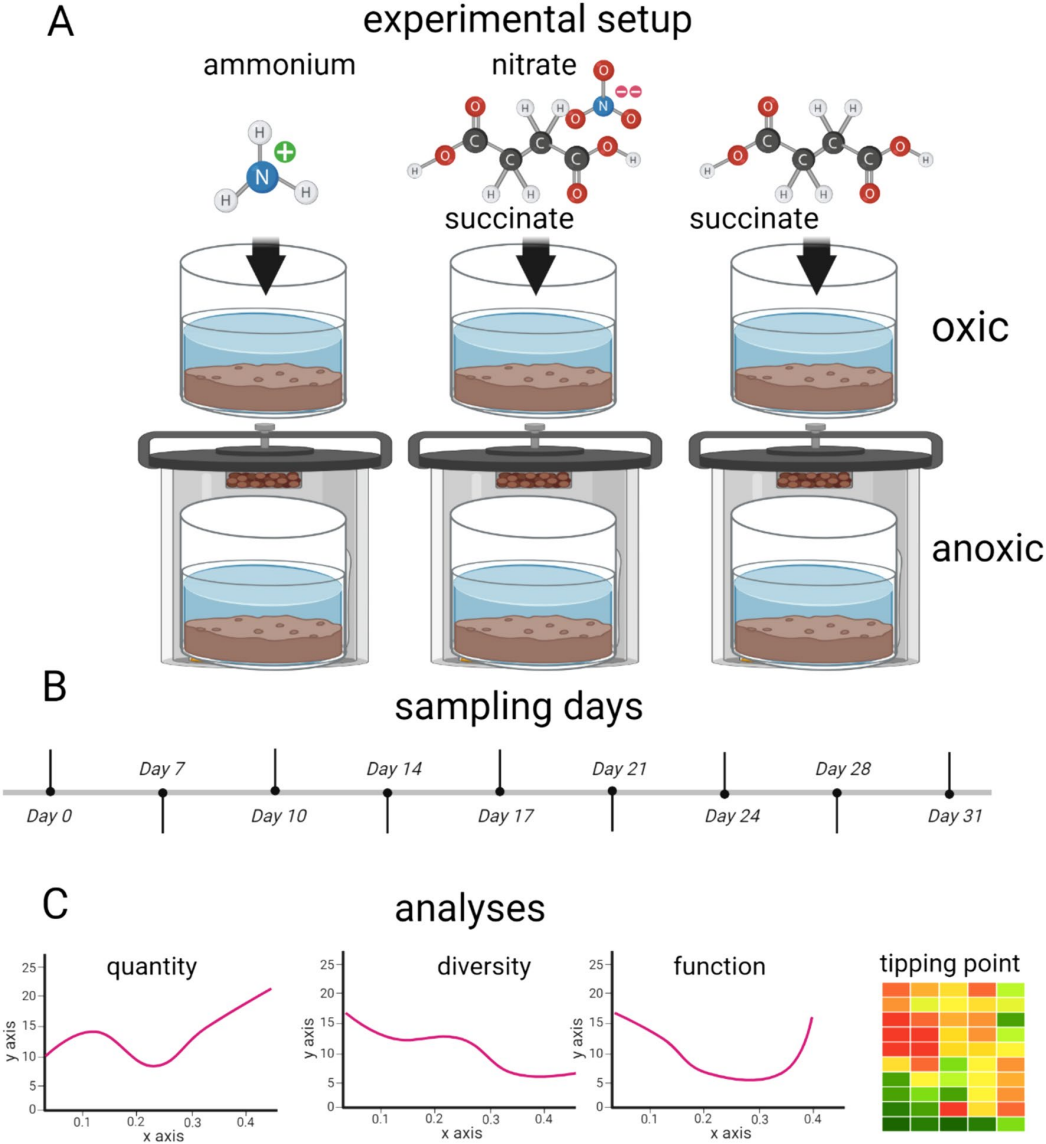
Experimental setup. **a** The experimental design included addition of ammonium, nitrate, and succinate to microcosm systems. Sulphate was added to all the samples. Incubations were done both oxic and anoxic. **b** Samples were collected over a period of 31 days. We used concentrations of 25 mM of both nitrate and succinate to simulate worst-case scenarios of point discharge of pollution. For ammonium, we used the optimal levels for ammonium-oxidizing archaea (AOA) without organic carbon, simulating an unpolluted environment. **c** The analytical strategies involved, quantification, analyses of diversity, and function, in addition to tipping point analyses

An outline of the experimental strategy is presented in Fig. 1.

## Materials and Methods

### Enrichment Media

The following common components were used in the specific enrichment media: 78g NaCl, 15 g MgSO47H2O, 15 g MgCl26H2O, 4.5 g CaCl2*H2O, 0.3 g KBr, and 3 L MilliRo water. The medium was subsequently autoclaved at 121 °C for a duration of 17 min. Once the autoclaving process was completed and the containers had sufficiently cooled, the following solutions were added by filtering through a 0.2-μm filter: 3 mL NaHCO3 [1M], 5 mL KH2PO4 [0.4 g/L], 1 mL FeNaEDTA solution [7.5 mM], 1 mL trace element solution (0.8 ml conc. HCl ~ 12.5 M), 3.0 mg H3BO3, 10.0 mg MnCl2·4H2O, 19.0 mg CoCl2·6H2O, 2.4 mg NiCl2·6H2O, 0.2 mg CuCl2·2H2O, 14.4 mg ZnSO4·7H2O, 3.6 mg Na2MoO4·2H2O, 99.2 ml deionized H2O, and 0.1 mL alpha ketoglutaric acid 100 mM. Subsequently, three distinct types of media were generated as follows:

- Ammonium-oxidizing medium, where 0.2 mL of 1 M NH4Cl was introduced aseptically to simulate nitrifying conditions.
- Nitrate-reducing medium, where 3 g of succinic acid and 2 g of NaNO3 were added under aseptic conditions to stimulate denitrification.
- Sulphate-reducing medium was established by aseptically adding 3 g of succinic acid to stimulate sulphate reduction.

### Microcosm Pollution Experiment

Sediment samples were collected from Hvitsten Bay in the Oslo fjord, Norway, during the spring of 2022, at a depth of three metres. The samples were collected with a 0.5 L Van Veen grab (Eijkelkamp, Netherland) and transported directly to the lab. Hundred gramme of sediment samples were then placed in 500-mL beakers along with 300 mL of specific enrichment medium (described above). The beakers were covered with aluminium foil and divided into two groups: one group was placed in a ten-litre anaerobic chamber with Anaerogen packs and indicators for anoxic conditions, while the other group was incubated under oxic conditions, exposed to air. All beakers were refrigerated at 10℃, to simulate the temperature in fjord water.

### Sample Collection and DNA Extraction

Three sediment subsamples from each beaker were collected every three or four days over the period of four weeks, resulting in a total of 144 samples. Approximately one gram of sediment was collected using a sterile spoon (Sarstedt, Germany) and placed in tubes containing 3 mL S.T.A.R buffer (Roche, Germany). The samples were frozen at − 20 °C.

DNA was extracted using the Zymo Research QuickDNA Fecal/Soil Microbe 96 MagBead kit, adhering to the guidelines provided by the manufacturer. Sediments (0.25 mL, resuspended in S.T.A.R. buffer) were added to BashingBead Lysis tubes with Lysis Solution and went through a mechanical lysis, using the TissueLyser II (Qiagen, Germany). Lysates were centrifuged to remove cellular debris and mineral components. DNA extraction was automated using the Kingfisher Flex robot for DNA extraction (Thermo-Fisher, USA). Eluted DNA was stored at – 20 ℃ for future use.

### Amplicon Sequencing

The 16S rRNA gene amplicon sequencing was carried out for bacteria following the method outlined in a prior study [1]. Briefly, the protocol included a first-stage PCR with a reaction mix containing HOT FIREPol® Blend Master Mix (Solis BioDyne Estland), Forward and Reverse primers (PRK341F and PRK806R) [37], template DNA, and nuclease-free water. A thermal cycler programme was set for 25 cycles of amplification. A manual purification of the PCR products was done using Sera Mag Speed Beads (Cytiva, USA). The purified DNA was utilized as template for a second-stage Index PCR step. The Index PCR reaction mix contained FirePol® Master Mix (Solis BioDyne), Forward and Reverse primers with unique indexes (F1-F8 and R1-R23), template DNA, and nuclease-free water. The programme consisted of 10 cycles of amplification. A two-stage PCR process was used to avoid PCR bias in the first stage due to the adapters. Samples were normalized based on DNA concentration. The pooled library underwent a final clean-up step using Sera Mag Speed Beads. Sequencing was performed on a MiSeq platform (Illumina, USA) using a V3 chemistry kit for 300 bp paired-end reads. The raw data are deposited in the Sequence Read Archive (SRA) under the accession number PRJNA1040446.

### Processing of Amplicon Sequencing Data

The sequencing data obtained were processed using the open-source tool VSEARCH 2.26 in R [29]. The sequencing reads underwent initial trimming, involving the removal of the first ten bases on the 3’ end, followed by merging. Unique sequences were retained, with those below a two-copy threshold being removed. Clustering of Operational Taxonomic Units (OTUs) was conducted, grouping 16S rRNA sequences that shared at least 97% similarity. Taxonomy was assigned using the RDP v18 database [11], with the sequence number per sample being rarified to 10,000 sequences, recommended by Robert Edgar [15]. The rationale for not using Dada2 [7] is that Dada2 handles high diversity sediment microbiota samples poorly (manuscript in preparation).

### Analyses of Amplicon Sequencing Data

Alpha and beta diversity metrics were derived using an OTU table that had been normalized through rarefaction to a depth of 10,000 sequences per sample. For the analysis of beta diversity, a Principal Coordinates Analysis (PCoA) plot was generated, employing Bray–Curtis dissimilarity metrics. To reveal distance decay patterns, changes in the read count per OTU were measured in relation to the initial sample [3]. This was done by summing the square of the relative change in permille for each OTU. Tipping point analyses were performed by analyzing the derivative of the read counts per OTU across the time series, with a peak indicating a tipping point [22]. The peak value for a tipping point was set empirically to absolute values above 10. Functional assignments were done using the Faprotax 1.2.7 database [21], using genus assignments from RDP as query for the functional assignment.

### qPCR Quantification

qPCR quantification of the 16S rRNA gene was done as previously described [1]. The qPCR reaction contained 1 × HOT FIREPol® EvaGreen® qPCR supermix (Solis BioDyne) and 0.2 μM of both Reverse (PRK806R) and Forward (PRK341F) primers [37]. The process of amplification was conducted using the C1000 Touch™ Thermal Cycler (manufactured by BioRad, USA). The quantification process was based on an established amplification efficiency of 1.8, and the determination of achieving 10^10 copies of the target amplicon as the Cq threshold.

### Statistical Analyses

Statistical analyses were done using either Matlab R2022b (MathWorks Inc, USA), or Minitab 18 (Minitab Inc, USA). The primary statistical method used for the time series data was the Kruskal–Wallis test, which provides non-parametric statistical significance testing when comparing differences among two or more groups and categories. This test was used to evaluate whether the values for each timepoint in the timeseries were significantly higher or lower than the overall median. The Kruskal–Wallis test was also used to assess the differences between oxic and anoxic conditions for each timepoint. The assessment of statistical significance for beta diversity was performed using Anosim, as implemented in the Fathom Toolbox for Matlab (www.usf.edu/marine-science/research/matlab-resources/index.aspx/). This is a non-parametric test to evaluate whether the groups are significantly different for pairwise distance or similarity data. The test statistic is based on the rank of similarities/dissimilarities within groups, as compared to between, represented by the R value, ranging between 1 and -1, with 1 indicating maximum dissimilarities between groups. P-values are derived by permutation tests. Advanced time series analyses were not applied due to the limited number of data points available for the time series data.

## Results

### Quantitative and Compositional Change of the Microbiota

**Fig. 2:**
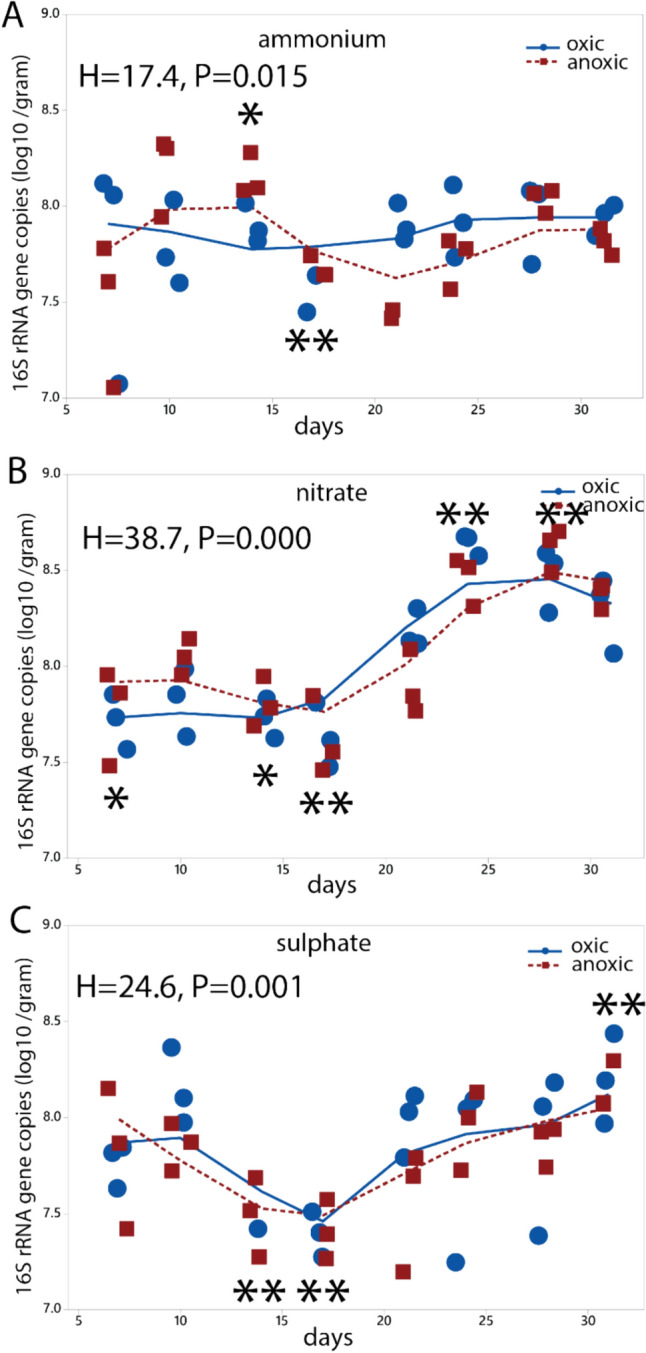
Quantity of 16S rRNA gene copies. The 16S rRNA gene copies were quantified using quantitative PCR. Quantification was done for the **a** the ammonium, **b** nitrate, and **c** sulphate experiments. Statistical testing was done using the Kruskal–Wallis test. Black asterisks represent the global test, with asterisk above the graphs indicating values significantly higher than the overall median, while asterisks below indicating values below the overall median. Green asterisks indicate a significant difference between oxic and anoxic incubation. Symbols: * p < 0.05, **, p < 0.01, ***. The numeric data are presented in Suppl. Table 1, and the statistical calculations in the Supplementary text p < 0.001.

**Fig. 3:**
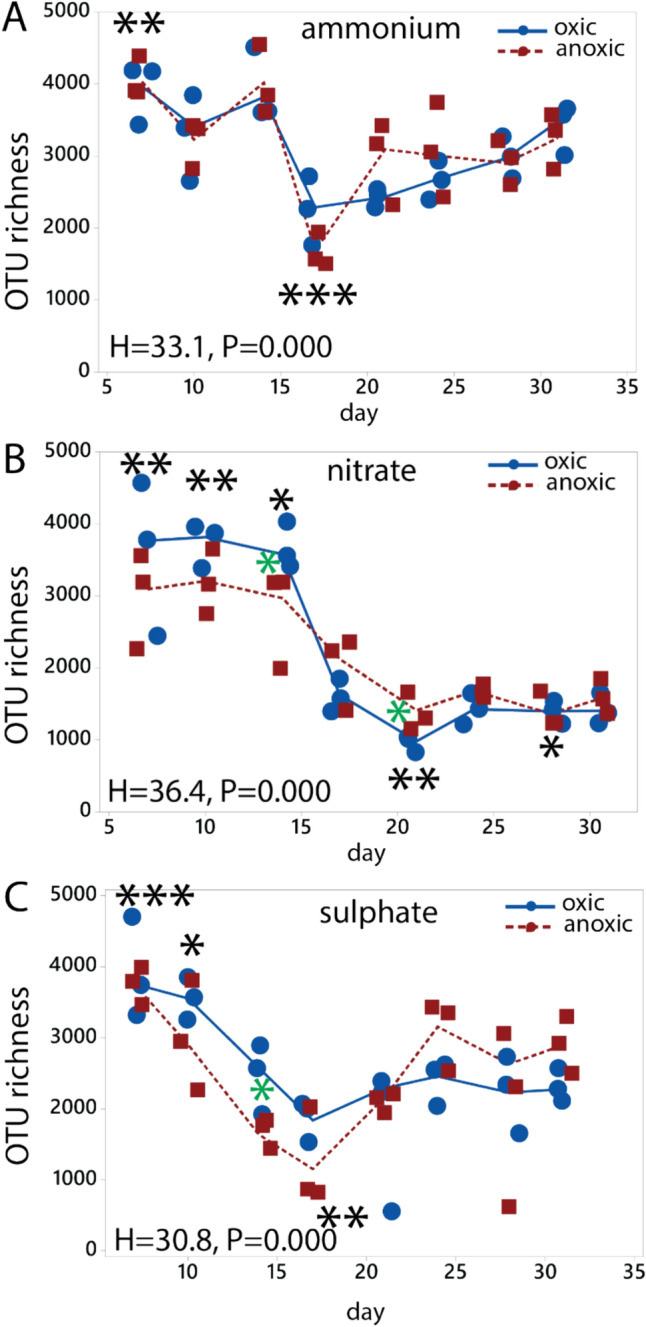
OTU richness distribution. The OTU richness was determined based on the number of observed OTUs. The number of OTUs was evaluated for **a** samples incubated with ammonium, **b** samples incubated with nitrate and **c** samples incubated with sulphate. Statistical testing was done using the Kruskal–Wallis test. Black asterisks represent the global test, with asterisks above the graphs indicate values significantly higher than the overall median, while asterisks below indicate values below the overall median. Green asterisks indicate a significant difference between oxic and anoxic incubation. Symbols: * p < 0.05, **, p < 0.01, *** p < 0.001. The numeric data are presented in Suppl. Table 1, and the statistical calculations in the Supplementary text

Quantitative 16S rRNA gene analysis indicated stable gene copy numbers for the ammonium incubation ranging from 7.7 to 8.1 log10 copy number per gram over the experimental period, as determined by the Kruskal–Wallis test, comparing the values for each timepoint with the overall median value (Fig. 2a). On the contrary, nitrate incubation exhibited a statistically significant (p < 0.01, Kruska–Wallis test) fivefold rise in copy numbers per gram, from 7.8 (log10) at day 17 to 8.6 at day 24 (Fig. 2b), while sulphate incubation displayed a modest decline in copy number, from 7.7 (log10) at day 7–7.3 at day 14 (p < 0.01), followed by an increase to 8.1 at day 31 (p < 0.01) (Fig. 2c). There were, however, no statistically significant differences between the oxic and anoxic incubations for the conditions evaluated. In terms of species richness, the samples supplemented with ammonium alone displayed the highest number of OTUs, with an average of 3137 ± 746 OTUs (mean ± std). Initially, there was a statistically significant decline in OTU richness between day 14 and 21 for ammonium (p < 0.001, Kruskal–Wallis test), but it subsequently increased until day 31 (Fig. 3a). The samples supplemented with sulphate exhibited lower OTU richness, averaging at 2512 ± 907 OTUs (mean ± std). The samples supplemented with nitrate showed the lowest OTU richness, averaging at 2184 ± 1014 OTUs (mean ± std). These samples also experienced a substantial decline in species richness (p < 0.01, Kruskal–Wallis test), with no apparent signs of recovery. The oxic condition shows a more rapid decline in species richness than the anoxic condition, with a statistically significant difference at day 14 (p < 0.05) (Fig. 3b). Notably, there was also a significant decrease in OTU richness between day 14 and 21 for the sulphate-supplemented samples (p < 0.01, < , Kruskal–Wallis test). Here, the anoxic condition showed a more pronounced decrease than the oxic condition (p < 0.05, Kruskal–Wallis test) (Fig. 3c).

**Fig. 4:**
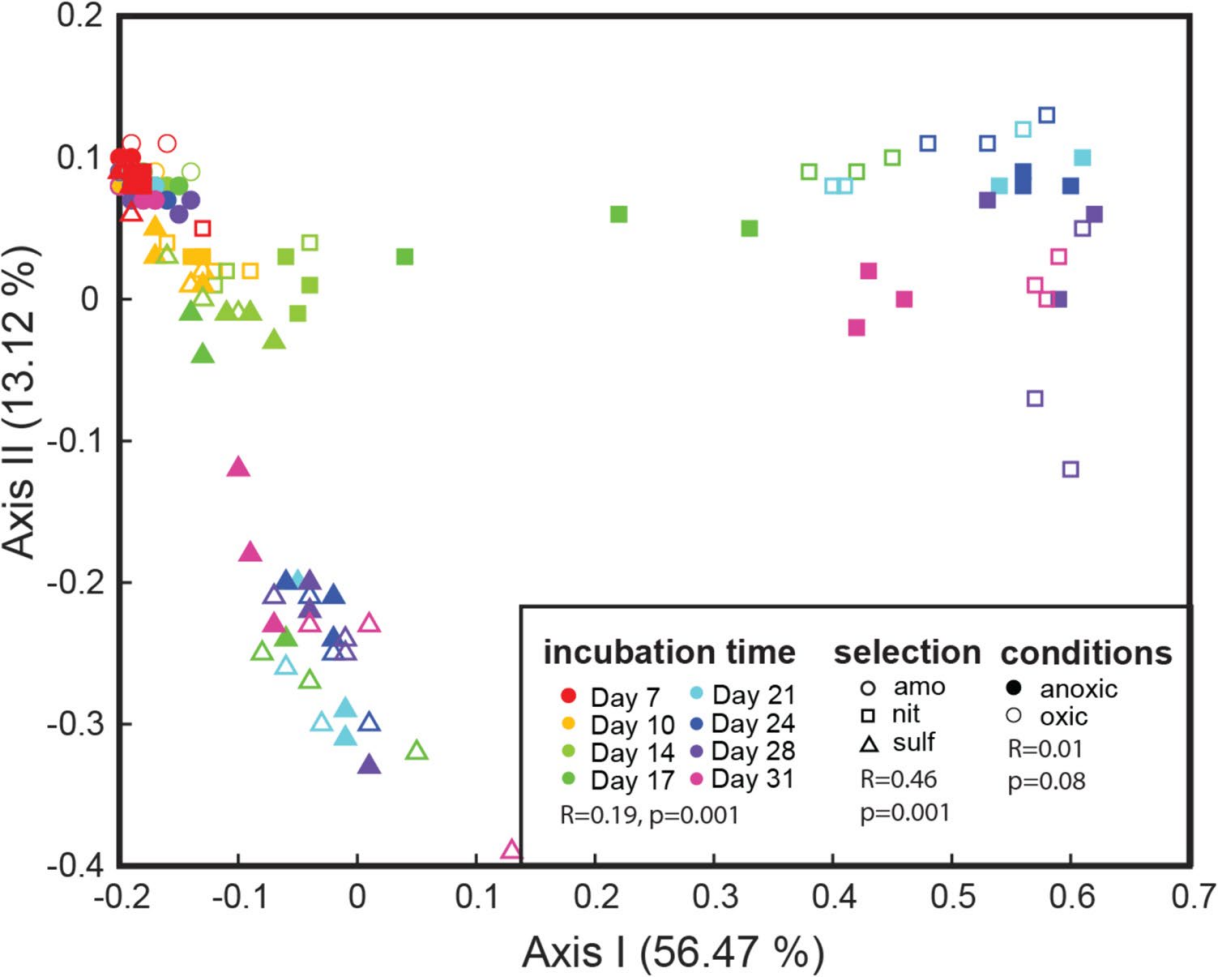
Beta diversity for the samples analysed. The beta diversity was determined using Bray Curtis distances and visualized using a PCoA plot. The two first PCoA axes are marked with the explained variance. Symbols are marked according to the different analytical factors, with the following abbreviations: panel A nit (nitrate), sulf (sulphate), ammo (ammonium); oxic (incubation under oxic conditions), anoxic (incubation under anoxic conditions); Incubation time (the numbers represent day after perturbation). The differences between the groups were evaluated using Anosim with 999 permutations

The measures for beta diversity confirmed the stability of the microbiota composition associated with ammonium, as visualized by the low spread in the PCoA plot (Fig. 4). However, the samples enriched with sulphate exhibited a different trajectory of change compared to the nitrate-enriched samples. There was a relatively large effect of the selection means, with R = 0.46 as determined by Anosims (*p* = 0.000, permutation test). Exposure to air did not show a statistically significant effect (*p* = 0.08). Time showed an overall statistically significant effect on beta diversity (*p* = 0.000, permutation test), but still lower effect (*R *= 0.19) than for selection means (Fig. 4c).

### Temporal Response of Microbiota Composition

**Fig. 5:**
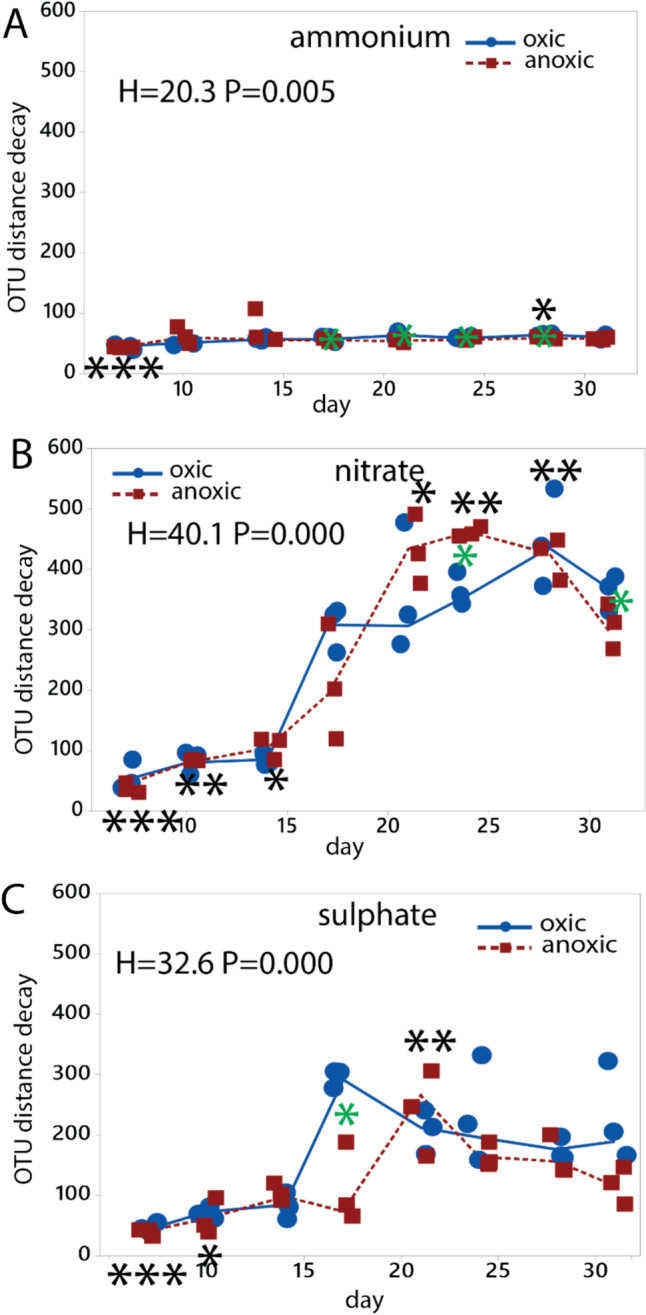
Distance decay pattern for the OTU composition. The distance decay pattern was determined relative to the OTU composition in the initial sediments. The decay pattern was evaluated for incubation with **a** ammonium, **b** nitrate, and **c** sulphate. Statistical testing was done using the Kruskal–Wallis test. Black asterisks represent the global test, with asterisk above the graphs indicate values significantly higher than the overall median, while asterisks below indicate values below the overall median. Green asterisks indicate a significant difference between oxic and anoxic incubation. Symbols: * p < 0.05, **, p < 0.01, *** p < 0.001. The numeric data are presented in Suppl. Table 1, and the statistical calculations in the Supplementary text

To investigate the temporal change of the microbiota, we conducted analyses to determine the OTU decay patterns for the various treatments. The first time point deviated from the rest of the time series for all conditions (Fig. 5). The samples treated with ammonium displayed minimal deviation, while there were still statistically significant differences between oxic and anoxic conditions (Fig. 5a). In contrast, the samples treated with nitrate exhibited the most significant response for the OTU decay pattern (Fig. 5b). There was also a statistically significant OTU decay for sulphate, with the samples exposed to air showing a more rapid decay in microbiota composition compared to the anoxically incubated samples (Fig. 5c).

**Fig. 6:**
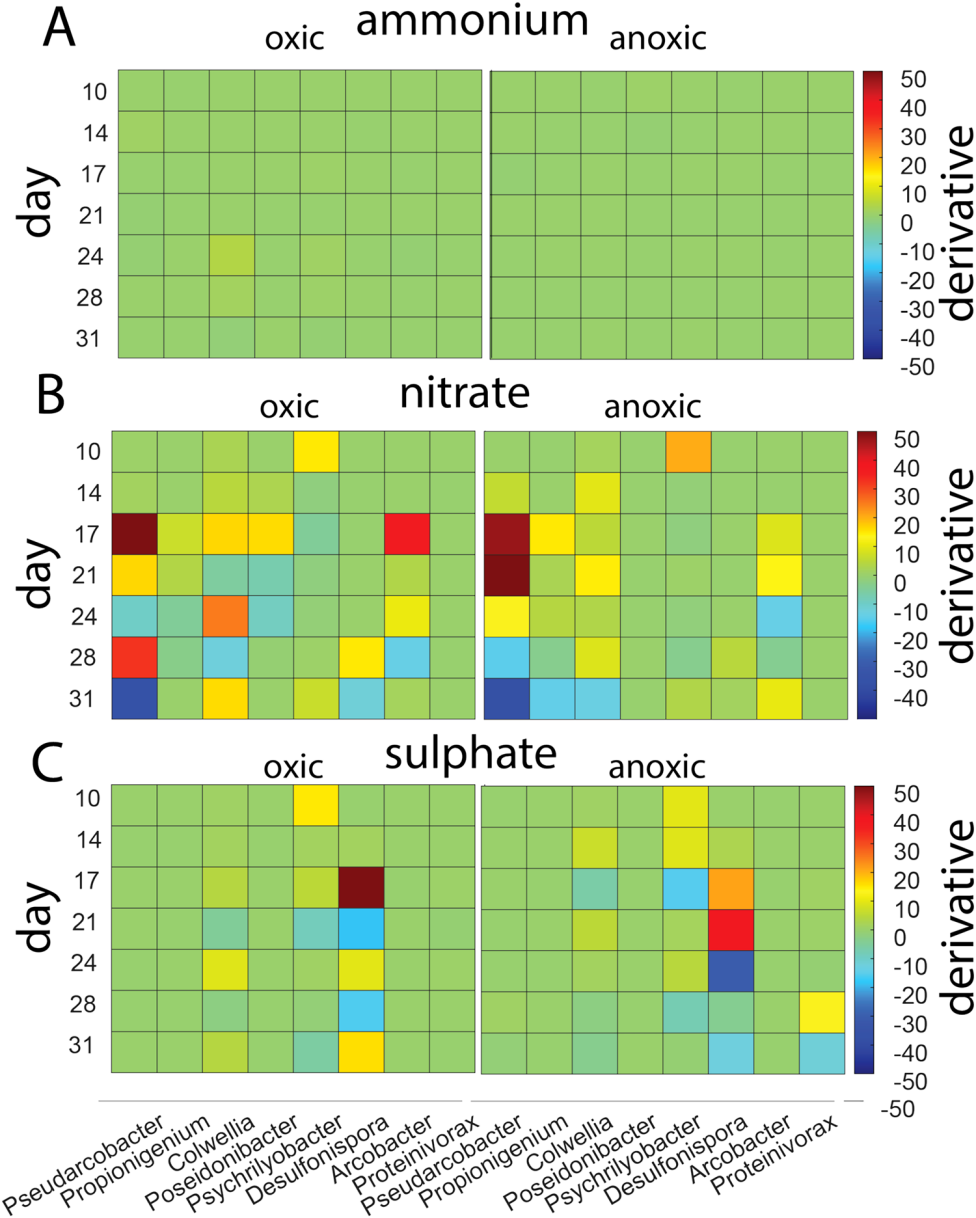
Genera showing a tipping point response. The tipping point analyses were determined by the derivative of read numbers for the OTU time series. Only genera showing an absolute response above 10 for the derivative are shown. The tipping points were evaluated for incubation with **a** ammonium, **b** nitrate, and **c** sulphate. Genera that display an absolute response above 10 are shown

Abrupt changes were also identified by analyzing the derivatives of the time series data. No significant responses were observed for samples with added ammonium (Fig. 6a). However, distinct and pronounced responses were observed between day 17 and 24 for samples with added nitrate and sulphate (Fig. 6b and c). Similar responses were observed for both oxic and anoxic conditions, although the responses were slightly delayed under anoxic conditions. The key changes were characterized by a sudden increase in *Pseudarcobacter* abundance for samples with added nitrate (Fig. 6b) and *Desulfonispora* abundance for sulphate (Fig. 6c). As samples contain sulphate with succinic acid, there was also a peak after 28 days for *Proteinivorax* under anoxic conditions (Fig. 6c).

### Inferred Shift in Microbiota Functions

**Fig. 7:**
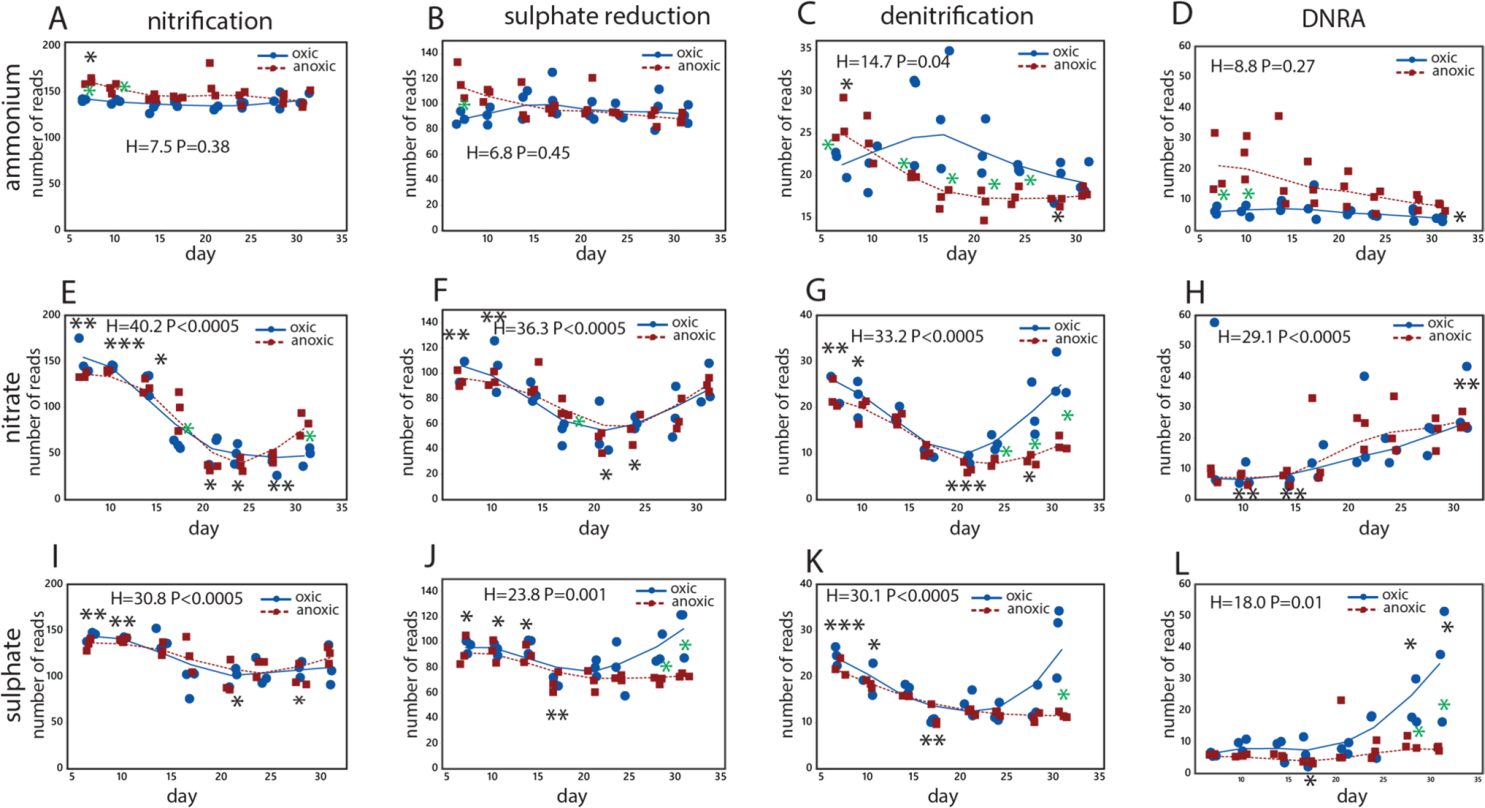
Inferred function. Functions inferred for the ammonium incubation experiment **a**–**d**, the nitrate incubation experiment **e**–**h**, and the sulphate incubation experiment **i**–**l**. The number of reads mapping to the respective functions (nitrification, sulphate reduction, denitrification, and DNRA) are shown. Statistical testing was done using the Kruskal–Wallis test. Black asterisks represent the global test, with asterisks above the graphs indicating values significantly higher than the overall median, while asterisks below indicate values below the overall median. Green asterisks indicate a significant difference between oxic and anoxic incubation. Symbols: * p < 0.05, ** p < 0.01, *** p < 0.001. The numeric data are presented in Suppl. Table 2, and the statistical calculations in the Supplementary text

The functional potential for nitrogen metabolism and sulphate reduction was inferred using the FAPROTAX database [21]. The ammonium incubation showed the overall lowest temporal response, with a slight decline in denitrification and DNRA (Fig. 7a–d). Nitrate addition, on the other hand, showed the largest response, with nitrification, sulphate reduction, and denitrification showing similar patterns, with a decline until about day 21, followed by a subsequent increase (Fig. 7e–g). Dissimilatory Nitrate Reduction to Ammonium (DNRA) was nearly absent until day 14, followed by an increase until the end of the experiment (Fig. 7h). For the sulphate addition experiment, there was a slight decrease in nitrification until day 21 (Fig. 7i). Under oxic conditions, there was an increase in sulphate reduction, denitrification, and DNRA from day 21 (Fig. 7j–l).

## Discussion

For the evaluation of pollution events, the shift in microbiota occurred abruptly between day 15 and 20, for both nitrate and sulphate pollution, resembling that of an ecological tipping point [12]. One challenge with identifying potential tipping points in nature is the time lag from the stressor to the actual response. Delayed responses could, therefore, be a contributing explanation as to why tipping points are rarely identified in natural ecosystems [18].

Addition of nitrate did by far show the most dramatic effect in our microcosm experiment. We detected a threefold decrease in OTU richness one month after the perturbation, in addition to a switch towards a Campylobacteriota-dominated microbiota. Marine Campylobacteriota are commonly associated with sulfide-rich habitats [8]. This may explain the delayed switch, since sulfide-rich environments would require prior sulphate reduction [25]. The switch towards Campylobacteriota was also associated with a major increase in cell density. At the genus level, *Pseudarchobacter* was the dominant Campylobacteriota genus detected in the current microcosm experiment. Metabolically, *Pseudarchobacter* is known as a sulfide-oxidizing and nitrate-reducing genus [36]. This supports the idea that the delay in response could be due to a demand for sulfide. While sulfide can serve as an electron donor in denitrification [9], it can also be a potent inhibitor of nitrous oxide reduction to nitrogen gas [30]. This highly specialized niche could potentially explain the drastic reduction in species richness observed for the nitrate microcosm experiment, which could potentially be linked to the availability of trace elements [27].

For microcosms containing sulphate, without added nitrate, the shift leaned towards a Firmicutes-dominated community. The main genus, *Desulfonispora*, is a spore former and a potential thiosulphate producer [13]. This may indicate that *Desulfonispora* is adapted to fluctuating environments, with specific triggers for spore germination. Such mechanisms have been extensively studied for the human gut microbiota [6]. A recent study has also unveiled that endospores in marine sediments could represent a large and unexplored diversity of microbes [35]. Thus, endospore germination could represent a source of unexpected responses in marine microbial ecology.

In the microcosms with ammonium-oxidizing medium, we only observed minor changes during the experimental period. Furthermore, there was no apparent difference between the samples incubated under oxic and anoxic conditions. Therefore, it is unlikely that we were able to induce ammonium oxidation since ammonium oxidation should have been favoured under oxic conditions. A plausible explanation is that the experimental period was too short. Enrichment of processes involving nitrification could potentially take months [26].

The impact of both oxic and anoxic conditions was modest, suggesting the significance of electron acceptors other than oxygen in the microcosm experiments [28]. The primary compositional contrast observed between oxic and anoxic environments was a notable rise in the presence of *Proteinivorax* at day 28 under anoxic conditions in the presence of sulphate. This shift could potentially signify a transition towards anaerobic proteolysis at the end of the experimental period [4].

The largest functional response was observed for the nitrate addition experiment. The inferred functions nitrification, sulphate reduction, and denitrification showed a minimum between two and three weeks, with a subsequent regain of the functions at the end of the experiment. The functional regain, however, was associated with a shift in microbiota composition, indicating functional but not taxonomic resilience. The decoupling of function and taxonomy has previously been suggested for marine microorganisms [20]. For sulphate, resilience was mainly observed under oxic conditions. This was surprising, as sulphate reduction and denitrification are anoxic processes. Recent evidence, however, suggests that the regain of growth can be associated with a structured environment [23], with oxygen gradients representing a potential structure. DNRA emerged late in the experiment, both for the nitrate and for the oxic sulphate addition experiment. In a continuous culture experiment, it has been shown that if nitrate is limiting with respect to organic carbon, then the nitrogen metabolism switches to DNRA to conserve the nitrogen (van den [32]). Thus, a potential explanation for the late increase in DNRA under our experimental conditions could be nitrogen limitation with respect to organic carbon.

In conclusion, our microcosm incubation experiments uncovered delayed, but abrupt compositional changes reminiscent of tipping points that are likely to be overlooked in natural environments. This underscores the importance of laboratory-based simulations in investigating marine ecological processes.

## Supplementary Information

Below is the link to the electronic supplementary material.

Supplementary file1 (DOCX 35 KB)

Supplementary file2 (DOCX 58 KB)

Supplementary file3 (DOCX 31 KB)
