# Supplementary material for: Delayed Shift in Microbiota Composition in a Marine Microcosm Pollution Experiment
Source: Curr Microbiol. 2024 Sep 18;81(11):365. doi: 10.1007/s00284-024-03869-5 (PMC11410848; doi:10.1007/s00284-024-03869-5)
Supplement: Supplementary file 1 — Supplementary file1 (DOCX 35 KB) [file 284_2024_3869_MOESM1_ESM.docx]

**Supplementary Table 2. Number of reads mapping to the differnt functions from the Faprotax database**

| **Selection** | **Incubation time (Days)** | **Condition** | **nitrification** | **sulphate reduction** | **DNRA** | **denitrification** |
| --- | --- | --- | --- | --- | --- | --- |
| ammonium | 7 | oxic | 166.32 | 133.60 | 32.13 | 29.03 |
| ammonium | 7 | oxic | 160.45 | 114.94 | 15.32 | 25.31 |
| ammonium | 7 | oxic | 159.30 | 105.71 | 12.77 | 24.73 |
| ammonium | 7 | anoxic | 141.06 | 88.93 | 5.61 | 22.23 |
| ammonium | 7 | anoxic | 142.79 | 83.31 | 5.74 | 20.12 |
| ammonium | 7 | anoxic | 142.32 | 95.08 | 7.71 | 22.77 |
| ammonium | 10 | oxic | 153.34 | 102.72 | 16.38 | 23.30 |
| ammonium | 10 | oxic | 157.53 | 110.01 | 25.57 | 27.40 |
| ammonium | 10 | oxic | 149.53 | 110.38 | 30.59 | 21.50 |
| ammonium | 10 | anoxic | 143.12 | 96.47 | 6.62 | 23.59 |
| ammonium | 10 | anoxic | 139.27 | 90.49 | 7.97 | 21.52 |
| ammonium | 10 | anoxic | 136.42 | 82.29 | 4.37 | 17.78 |
| ammonium | 14 | oxic | 148.02 | 116.60 | 38.17 | 20.41 |
| ammonium | 14 | oxic | 141.73 | 88.08 | 8.91 | 19.66 |
| ammonium | 14 | oxic | 142.52 | 91.73 | 12.80 | 19.62 |
| ammonium | 14 | anoxic | 134.03 | 106.48 | 8.73 | 30.97 |
| ammonium | 14 | anoxic | 126.81 | 87.57 | 6.09 | 21.33 |
| ammonium | 14 | anoxic | 139.27 | 110.66 | 9.11 | 31.76 |
| ammonium | 17 | oxic | 141.36 | 96.89 | 12.77 | 17.80 |
| ammonium | 17 | oxic | 143.48 | 93.62 | 8.06 | 17.57 |
| ammonium | 17 | oxic | 146.56 | 100.04 | 23.21 | 16.51 |
| ammonium | 17 | anoxic | 141.51 | 125.77 | 14.22 | 35.20 |
| ammonium | 17 | anoxic | 134.98 | 92.12 | 4.04 | 21.22 |
| ammonium | 17 | anoxic | 139.09 | 102.05 | 7.26 | 26.60 |
| ammonium | 21 | oxic | 180.86 | 120.29 | 8.38 | 14.65 |
| ammonium | 21 | oxic | 146.60 | 96.40 | 18.89 | 17.03 |
| ammonium | 21 | oxic | 153.17 | 92.29 | 14.39 | 17.90 |
| ammonium | 21 | anoxic | 130.27 | 88.13 | 6.27 | 22.47 |
| ammonium | 21 | anoxic | 131.70 | 90.30 | 5.51 | 20.65 |
| ammonium | 21 | anoxic | 135.89 | 102.84 | 6.71 | 26.29 |
| ammonium | 24 | oxic | 146.70 | 92.35 | 10.44 | 18.44 |
| ammonium | 24 | oxic | 150.24 | 91.53 | 5.97 | 16.88 |
| ammonium | 24 | oxic | 136.36 | 94.56 | 13.58 | 17.00 |
| ammonium | 24 | anoxic | 137.36 | 91.56 | 4.22 | 20.93 |
| ammonium | 24 | anoxic | 137.07 | 100.29 | 5.82 | 21.55 |
| ammonium | 24 | anoxic | 132.23 | 88.71 | 4.35 | 20.64 |
| ammonium | 28 | oxic | 137.75 | 83.04 | 6.28 | 16.54 |
| ammonium | 28 | oxic | 148.83 | 94.98 | 10.54 | 17.61 |
| ammonium | 28 | oxic | 142.77 | 89.89 | 10.77 | 16.74 |
| ammonium | 28 | anoxic | 132.94 | 79.97 | 3.45 | 16.26 |
| ammonium | 28 | anoxic | 139.44 | 98.42 | 5.81 | 22.05 |
| ammonium | 28 | anoxic | 150.66 | 112.92 | 6.66 | 20.12 |
| ammonium | 31 | oxic | 151.79 | 93.10 | 6.43 | 18.28 |
| ammonium | 31 | oxic | 132.34 | 85.46 | 8.61 | 17.22 |
| ammonium | 31 | oxic | 138.35 | 87.23 | 8.75 | 17.86 |
| ammonium | 31 | anoxic | 139.16 | 85.04 | 3.51 | 17.69 |
| ammonium | 31 | anoxic | 137.25 | 89.98 | 4.21 | 18.23 |
| ammonium | 31 | anoxic | 148.04 | 99.04 | 3.63 | 22.06 |
| nitrate | 7 | oxic | 136.46 | 92.49 | 4.70 | 20.72 |
| nitrate | 7 | oxic | 133.81 | 99.93 | 7.26 | 27.43 |
| nitrate | 7 | oxic | 131.10 | 93.98 | 10.81 | 21.58 |
| nitrate | 7 | anoxic | 143.21 | 109.68 | 7.01 | 27.97 |
| nitrate | 7 | anoxic | 147.03 | 95.35 | 5.74 | 19.88 |
| nitrate | 7 | anoxic | 175.08 | 200.55 | 58.30 | 59.47 |
| nitrate | 10 | oxic | 142.58 | 100.33 | 5.32 | 20.88 |
| nitrate | 10 | oxic | 138.28 | 92.29 | 7.26 | 16.99 |
| nitrate | 10 | oxic | 138.60 | 90.91 | 7.33 | 20.66 |
| nitrate | 10 | anoxic | 142.79 | 87.03 | 5.01 | 18.55 |
| nitrate | 10 | anoxic | 148.46 | 122.86 | 12.07 | 25.83 |
| nitrate | 10 | anoxic | 148.79 | 102.41 | 5.32 | 23.33 |
| nitrate | 14 | oxic | 132.59 | 105.00 | 9.82 | 18.31 |
| nitrate | 14 | oxic | 119.40 | 85.71 | 5.71 | 18.21 |
| nitrate | 14 | oxic | 116.48 | 82.68 | 6.97 | 16.34 |
| nitrate | 14 | anoxic | 116.16 | 79.09 | 4.44 | 16.77 |
| nitrate | 14 | anoxic | 136.98 | 90.71 | 5.24 | 19.66 |
| nitrate | 14 | anoxic | 132.84 | 80.11 | 6.30 | 17.41 |
| nitrate | 17 | oxic | 73.32 | 78.13 | 34.01 | 9.89 |
| nitrate | 17 | oxic | 115.67 | 67.96 | 9.01 | 11.13 |
| nitrate | 17 | oxic | 103.43 | 71.05 | 8.28 | 9.94 |
| nitrate | 17 | anoxic | 62.20 | 45.46 | 8.12 | 10.80 |
| nitrate | 17 | anoxic | 63.78 | 54.88 | 12.62 | 9.90 |
| nitrate | 17 | anoxic | 54.87 | 56.21 | 16.58 | 8.47 |
| nitrate | 21 | oxic | 38.50 | 52.45 | 25.31 | 7.79 |
| nitrate | 21 | oxic | 28.91 | 40.44 | 17.52 | 7.40 |
| nitrate | 21 | oxic | 35.81 | 52.32 | 26.14 | 6.96 |
| nitrate | 21 | anoxic | 62.38 | 47.56 | 14.35 | 7.96 |
| nitrate | 21 | anoxic | 36.34 | 37.97 | 11.61 | 7.05 |
| nitrate | 21 | anoxic | 63.12 | 80.34 | 39.05 | 9.75 |
| nitrate | 24 | oxic | 37.29 | 45.31 | 14.89 | 6.73 |
| nitrate | 24 | oxic | 43.85 | 57.64 | 20.31 | 7.88 |
| nitrate | 24 | oxic | 33.99 | 64.07 | 32.35 | 9.73 |
| nitrate | 24 | anoxic | 57.35 | 61.45 | 12.43 | 14.72 |
| nitrate | 24 | anoxic | 53.49 | 62.70 | 21.07 | 11.03 |
| nitrate | 24 | anoxic | 40.44 | 53.84 | 14.81 | 11.24 |
| nitrate | 28 | oxic | 52.17 | 52.22 | 14.60 | 6.44 |
| nitrate | 28 | oxic | 38.14 | 63.27 | 23.49 | 7.80 |
| nitrate | 28 | oxic | 44.79 | 78.65 | 27.80 | 9.07 |
| nitrate | 28 | anoxic | 25.14 | 46.98 | 15.13 | 13.71 |
| nitrate | 28 | anoxic | 38.08 | 68.04 | 22.12 | 17.71 |
| nitrate | 28 | anoxic | 49.92 | 86.69 | 24.42 | 24.42 |
| nitrate | 31 | oxic | 91.77 | 95.73 | 24.75 | 14.61 |
| nitrate | 31 | oxic | 86.04 | 87.85 | 27.46 | 11.67 |
| nitrate | 31 | oxic | 70.62 | 90.62 | 24.69 | 12.26 |
| nitrate | 31 | anoxic | 39.84 | 106.76 | 44.49 | 32.72 |
| nitrate | 31 | anoxic | 53.55 | 82.81 | 25.71 | 24.49 |
| nitrate | 31 | anoxic | 58.38 | 78.45 | 23.38 | 22.30 |
| sulphate | 7 | oxic | 142.23 | 104.05 | 5.29 | 24.12 |
| sulphate | 7 | oxic | 125.84 | 83.50 | 5.72 | 21.73 |
| sulphate | 7 | oxic | 135.54 | 90.04 | 5.21 | 19.97 |
| sulphate | 7 | anoxic | 146.15 | 98.70 | 7.02 | 24.09 |
| sulphate | 7 | anoxic | 139.36 | 89.63 | 4.80 | 22.23 |
| sulphate | 7 | anoxic | 145.45 | 101.02 | 4.92 | 26.35 |
| sulphate | 10 | oxic | 138.26 | 92.65 | 6.63 | 18.59 |
| sulphate | 10 | oxic | 143.40 | 99.92 | 4.67 | 19.35 |
| sulphate | 10 | oxic | 136.45 | 84.57 | 4.97 | 17.39 |
| sulphate | 10 | anoxic | 140.27 | 89.67 | 10.22 | 19.50 |
| sulphate | 10 | anoxic | 141.47 | 93.36 | 10.46 | 16.00 |
| sulphate | 10 | anoxic | 142.30 | 99.56 | 8.17 | 22.30 |
| sulphate | 14 | oxic | 121.63 | 87.71 | 4.98 | 15.39 |
| sulphate | 14 | oxic | 138.51 | 97.64 | 4.91 | 15.58 |
| sulphate | 14 | oxic | 127.87 | 85.09 | 4.74 | 15.71 |
| sulphate | 14 | anoxic | 154.04 | 90.64 | 3.95 | 18.40 |
| sulphate | 14 | anoxic | 135.46 | 100.06 | 9.04 | 16.70 |
| sulphate | 14 | anoxic | 129.59 | 101.97 | 10.45 | 17.38 |
| sulphate | 17 | oxic | 104.24 | 61.41 | 2.63 | 10.26 |
| sulphate | 17 | oxic | 142.62 | 74.90 | 4.27 | 14.25 |
| sulphate | 17 | oxic | 123.48 | 68.01 | 3.04 | 10.07 |
| sulphate | 17 | anoxic | 103.62 | 65.85 | 5.53 | 11.19 |
| sulphate | 17 | anoxic | 76.50 | 71.75 | 11.51 | 10.25 |
| sulphate | 17 | anoxic | 101.37 | 65.79 | 2.82 | 10.20 |
| sulphate | 21 | oxic | 85.94 | 78.71 | 23.21 | 12.69 |
| sulphate | 21 | oxic | 87.74 | 59.28 | 4.42 | 11.28 |
| sulphate | 21 | oxic | 116.81 | 70.14 | 3.85 | 12.73 |
| sulphate | 21 | anoxic | 89.46 | 78.84 | 8.58 | 13.89 |
| sulphate | 21 | anoxic | 109.84 | 84.26 | 8.67 | 16.74 |
| sulphate | 21 | anoxic | 103.18 | 71.30 | 7.25 | 11.27 |
| sulphate | 24 | oxic | 97.37 | 70.85 | 5.21 | 11.76 |
| sulphate | 24 | oxic | 114.92 | 72.20 | 11.11 | 12.19 |
| sulphate | 24 | oxic | 114.21 | 74.64 | 7.33 | 12.48 |
| sulphate | 24 | anoxic | 99.44 | 78.83 | 18.56 | 11.12 |
| sulphate | 24 | anoxic | 119.67 | 101.00 | 17.29 | 14.57 |
| sulphate | 24 | anoxic | 91.49 | 58.31 | 4.46 | 10.00 |
| sulphate | 28 | oxic | 89.85 | 72.87 | 11.32 | 12.38 |
| sulphate | 28 | oxic | 114.91 | 69.84 | 7.39 | 10.63 |
| sulphate | 28 | oxic | 93.07 | 66.07 | 9.16 | 9.76 |
| sulphate | 28 | anoxic | 97.14 | 106.36 | 29.56 | 18.12 |
| sulphate | 28 | anoxic | 117.17 | 87.47 | 17.68 | 12.07 |
| sulphate | 28 | anoxic | 110.36 | 86.36 | 17.58 | 11.94 |
| sulphate | 31 | oxic | 115.54 | 74.17 | 8.03 | 12.16 |
| sulphate | 31 | oxic | 126.64 | 72.62 | 7.32 | 11.62 |
| sulphate | 31 | oxic | 133.07 | 76.71 | 7.07 | 11.33 |
| sulphate | 31 | anoxic | 91.58 | 121.69 | 51.12 | 31.32 |
| sulphate | 31 | anoxic | 134.23 | 86.69 | 15.53 | 20.22 |
| sulphate | 31 | anoxic | 107.42 | 120.44 | 38.40 | 33.83 |
