# Supplementary material for: Delayed Shift in Microbiota Composition in a Marine Microcosm Pollution Experiment
Source: Curr Microbiol. 2024 Sep 18;81(11):365. doi: 10.1007/s00284-024-03869-5 (PMC11410848; doi:10.1007/s00284-024-03869-5)
Supplement: Supplementary file 2 — Supplementary file2 (DOCX 58 KB) [file 284_2024_3869_MOESM2_ESM.docx]

Supplementary statistical calculations

Kruskal-Wallis Test: copynumber vs day

Ammonium

| Day | N | Median | Mean Rank | Z-Value |
| --- | --- | --- | --- | --- |
| 7 | 6 | 7.70527 | 19.7 | -0.90 |
| 10 | 6 | 7.99034 | 30.7 | 1.15 |
| 14 | 6 | 8.05723 | 36.2 | 2.18 |
| 17 | 6 | 7.65104 | 10.0 | -2.71 |
| 21 | 6 | 7.65001 | 16.3 | -1.53 |
| 24 | 6 | 7.77369 | 23.2 | -0.25 |
| 28 | 6 | 8.07648 | 34.0 | 1.78 |
| 31 | 6 | 7.87214 | 26.0 | 0.28 |
| Overall | 48 |  | 24.5 |  |

Test

| Null hypothesis | H₀: All medians are equal |
| --- | --- |
| Alternative hypothesis | H₁: At least one median is different |

| DF | H-Value | P-Value |
| --- | --- | --- |
| 7 | 17.41 | 0.015 |

Kruskal-Wallis Test: copynumber vs day

Nitrate

| Day | N | Median | Mean Rank | Z-Value |
| --- | --- | --- | --- | --- |
| 7 | 6 | 7.77763 | 12.8 | -2.18 |
| 10 | 6 | 7.98772 | 21.0 | -0.65 |
| 14 | 6 | 7.74672 | 12.3 | -2.28 |
| 17 | 6 | 7.56955 | 7.7 | -3.15 |
| 21 | 6 | 8.09962 | 24.5 | 0.00 |
| 24 | 6 | 8.55321 | 41.7 | 3.21 |
| 28 | 6 | 8.53895 | 41.5 | 3.18 |
| 31 | 6 | 8.40736 | 34.5 | 1.87 |
| Overall | 48 |  | 24.5 |  |

Test

| Null hypothesis | H₀: All medians are equal |
| --- | --- |
| Alternative hypothesis | H₁: At least one median is different |

| DF | H-Value | P-Value |
| --- | --- | --- |
| 7 | 38.68 | 0.000 |

Kruskal-Wallis Test: copynumber vs day

Sulphate

| Day | N | Median | Mean Rank | Z-Value |
| --- | --- | --- | --- | --- |
| 7 | 6 | 7.83117 | 24.0 | -0.09 |
| 10 | 6 | 7.96210 | 32.7 | 1.53 |
| 14 | 6 | 7.34289 | 8.2 | -3.06 |
| 17 | 6 | 7.37292 | 9.7 | -2.77 |
| 21 | 6 | 7.82477 | 24.8 | 0.06 |
| 24 | 6 | 8.02419 | 29.2 | 0.87 |
| 28 | 6 | 7.90865 | 28.5 | 0.75 |
| 31 | 6 | 8.13906 | 39.0 | 2.71 |
| Overall | 48 |  | 24.5 |  |

Test

| Null hypothesis | H₀: All medians are equal |
| --- | --- |
| Alternative hypothesis | H₁: At least one median is different |

| Method | DF | H-Value | P-Value |
| --- | --- | --- | --- |
| Not adjusted for ties | 7 | 24.55 | 0.001 |
| Adjusted for ties | 7 | 24.55 | 0.001 |

Kruskal-Wallis Test: species richness vs day

Ammonium

| DayNum | N | Median | Mean Rank | Z-Value |
| --- | --- | --- | --- | --- |
| 7 | 6 | 3984.0 | 41.3 | 3.15 |
| 10 | 6 | 3380.5 | 27.0 | 0.47 |
| 14 | 6 | 3776.0 | 40.2 | 2.93 |
| 17 | 6 | 1820.0 | 5.3 | -3.59 |
| 21 | 6 | 2516.0 | 14.8 | -1.81 |
| 24 | 6 | 2784.5 | 18.3 | -1.15 |
| 28 | 6 | 2941.5 | 19.7 | -0.90 |
| 31 | 6 | 3441.5 | 29.3 | 0.90 |
| Overall | 48 |  | 24.5 |  |

Test

| Null hypothesis | H₀: All medians are equal |
| --- | --- |
| Alternative hypothesis | H₁: At least one median is different |

| DF | H-Value | P-Value |
| --- | --- | --- |
| 7 | 33.08 | 0.000 |

Kruskal-Wallis Test: species richness vs day

Nitrate

| DayNum | N | Median | Mean Rank | Z-Value |
| --- | --- | --- | --- | --- |
| 7 | 6 | 3351.5 | 39.0 | 2.71 |
| 10 | 6 | 3578.5 | 40.7 | 3.02 |
| 14 | 6 | 3277.5 | 38.2 | 2.56 |
| 17 | 6 | 1763.0 | 23.0 | -0.28 |
| 21 | 6 | 1148.5 | 7.8 | -3.12 |
| 24 | 6 | 1658.0 | 19.0 | -1.03 |
| 28 | 6 | 1343.0 | 12.3 | -2.28 |
| 31 | 6 | 1456.0 | 16.0 | -1.59 |
| Overall | 48 |  | 24.5 |  |

Test

| Null hypothesis | H₀: All medians are equal |
| --- | --- |
| Alternative hypothesis | H₁: At least one median is different |

| DF | H-Value | P-Value |
| --- | --- | --- |
| 7 | 36.40 | 0.000 |

Kruskal-Wallis Test: species richness vs day

Sulphate

| DayNum | N | Median | Mean Rank | Z-Value |
| --- | --- | --- | --- | --- |
| 7 | 6 | 3728.5 | 43.7 | 3.59 |
| 10 | 6 | 3397.5 | 37.2 | 2.37 |
| 14 | 6 | 1878.5 | 16.2 | -1.56 |
| 17 | 6 | 1714.0 | 8.2 | -3.06 |
| 21 | 6 | 2171.0 | 15.3 | -1.71 |
| 24 | 6 | 2659.0 | 30.2 | 1.06 |
| 28 | 6 | 2300.5 | 19.5 | -0.94 |
| 31 | 6 | 2461.5 | 25.8 | 0.25 |
| Overall | 48 |  | 24.5 |  |

Test

| Null hypothesis | H₀: All medians are equal |
| --- | --- |
| Alternative hypothesis | H₁: At least one median is different |

| DF | H-Value | P-Value |
| --- | --- | --- |
| 7 | 30.82 | 0.000 |

Kruskal-Wallis Test: Time decay vs Date

Ammonium

| Date | N | Median | Mean Rank | Z-Value |
| --- | --- | --- | --- | --- |
| 7 | 6 | 44.0642 | 3.7 | -3.90 |
| 10 | 6 | 52.2820 | 20.5 | -0.75 |
| 14 | 6 | 57.9264 | 29.7 | 0.97 |
| 17 | 6 | 55.6247 | 21.5 | -0.56 |
| 21 | 6 | 57.5071 | 27.8 | 0.62 |
| 24 | 6 | 57.3036 | 28.2 | 0.69 |
| 28 | 6 | 60.3987 | 36.2 | 2.18 |
| 31 | 6 | 57.9774 | 28.5 | 0.75 |
| Overall | 48 |  | 24.5 |  |

Test

| Null hypothesis | H₀: All medians are equal |
| --- | --- |
| Alternative hypothesis | H₁: At least one median is different |

| DF | H-Value | P-Value |
| --- | --- | --- |
| 7 | 20.28 | 0.005 |

Kruskal-Wallis Test: Time decay vs Date

Nitrate

| Date | N | Median | Mean Rank | Z-Value |
| --- | --- | --- | --- | --- |
| 7 | 6 | 43.308 | 3.8 | -3.87 |
| 10 | 6 | 80.412 | 10.7 | -2.59 |
| 14 | 6 | 95.098 | 14.0 | -1.96 |
| 17 | 6 | 288.581 | 23.2 | -0.25 |
| 21 | 6 | 406.907 | 36.3 | 2.21 |
| 24 | 6 | 428.440 | 38.5 | 2.62 |
| 28 | 6 | 433.034 | 40.2 | 2.93 |
| 31 | 6 | 341.616 | 29.3 | 0.90 |
| Overall | 48 |  | 24.5 |  |

Test

| Null hypothesis | H₀: All medians are equal |
| --- | --- |
| Alternative hypothesis | H₁: At least one median is different |

| DF | H-Value | P-Value |
| --- | --- | --- |
| 7 | 40.88 | 0.000 |

Kruskal-Wallis Test: Time decay vs Date

Sulphate

| Date | N | Median | Mean Rank | Z-Value |
| --- | --- | --- | --- | --- |
| 7 | 6 | 42.007 | 3.8 | -3.87 |
| 10 | 6 | 65.766 | 11.5 | -2.43 |
| 14 | 6 | 89.072 | 16.5 | -1.50 |
| 17 | 6 | 227.668 | 31.7 | 1.34 |
| 21 | 6 | 227.287 | 38.8 | 2.68 |
| 24 | 6 | 172.887 | 33.8 | 1.75 |
| 28 | 6 | 164.202 | 30.5 | 1.12 |
| 31 | 6 | 152.457 | 29.3 | 0.90 |
| Overall | 48 |  | 24.5 |  |

Test

| Null hypothesis | H₀: All medians are equal |
| --- | --- |
| Alternative hypothesis | H₁: At least one median is different |

| DF | H-Value | P-Value |
| --- | --- | --- |
| 7 | 32.55 | 0.000 |

Kruskal-Wallis Test: Nitrification vs Date

Sulphate

Descriptive Statistics

| DayNum | N | Median | Mean Rank | Z-Value |
| --- | --- | --- | --- | --- |
| 7 | 6 | 140.796 | 39.5 | 2.81 |
| 10 | 6 | 140.871 | 40.3 | 2.96 |
| 14 | 6 | 132.526 | 34.5 | 1.87 |
| 17 | 6 | 103.929 | 18.8 | -1.06 |
| 21 | 6 | 96.321 | 10.3 | -2.65 |
| 24 | 6 | 106.824 | 15.3 | -1.71 |
| 28 | 6 | 103.750 | 14.0 | -1.96 |
| 31 | 6 | 121.086 | 23.2 | -0.25 |
| Overall | 48 |  | 24.5 |  |

Test

| Null hypothesis | H₀: All medians are equal |
| --- | --- |
| Alternative hypothesis | H₁: At least one median is different |

| DF | H-Value | P-Value |
| --- | --- | --- |
| 7 | 30.75 | 0.000 |

Kruskal-Wallis Test: Sulphate reduction vs Date

Sulphate

Descriptive Statistics

| DayNum | N | Median | Mean Rank | Z-Value |
| --- | --- | --- | --- | --- |
| 7 | 6 | 94.3655 | 35.5 | 2.06 |
| 10 | 6 | 93.0060 | 34.5 | 1.87 |
| 14 | 6 | 94.1393 | 35.3 | 2.03 |
| 17 | 6 | 66.9271 | 8.2 | -3.06 |
| 21 | 6 | 75.0030 | 14.7 | -1.84 |
| 24 | 6 | 73.4224 | 17.3 | -1.34 |
| 28 | 6 | 79.6141 | 21.8 | -0.50 |
| 31 | 6 | 81.7019 | 28.7 | 0.78 |
| Overall | 48 |  | 24.5 |  |

Test

| Null hypothesis | H₀: All medians are equal |
| --- | --- |
| Alternative hypothesis | H₁: At least one median is different |

| DF | H-Value | P-Value |
| --- | --- | --- |
| 7 | 23.81 | 0.001 |

Kruskal-Wallis Test: Denitrification vs Date

Sulphate

Descriptive Statistics

| DayNum | N | Median | Mean Rank | Z-Value |
| --- | --- | --- | --- | --- |
| 7 | 6 | 23.1604 | 42.8 | 3.43 |
| 10 | 6 | 18.9690 | 36.0 | 2.15 |
| 14 | 6 | 16.2040 | 29.7 | 0.97 |
| 17 | 6 | 10.2560 | 8.5 | -2.99 |
| 21 | 6 | 12.7078 | 19.7 | -0.90 |
| 24 | 6 | 11.9724 | 14.5 | -1.87 |
| 28 | 6 | 12.0031 | 15.3 | -1.71 |
| 31 | 6 | 16.1898 | 29.5 | 0.94 |
| Overall | 48 |  | 24.5 |  |

Test

| Null hypothesis | H₀: All medians are equal |
| --- | --- |
| Alternative hypothesis | H₁: At least one median is different |

| DF | H-Value | P-Value |
| --- | --- | --- |
| 7 | 30.11 | 0.000 |

Kruskal-Wallis Test: DNRA vs Date

Sulphate

Descriptive Statistics

| DayNum | N | Median | Mean Rank | Z-Value |
| --- | --- | --- | --- | --- |
| 7 | 6 | 5.2524 | 16.7 | -1.47 |
| 10 | 6 | 7.4013 | 23.8 | -0.12 |
| 14 | 6 | 4.9473 | 18.2 | -1.18 |
| 17 | 6 | 3.6568 | 11.7 | -2.40 |
| 21 | 6 | 7.9122 | 23.5 | -0.19 |
| 24 | 6 | 9.2209 | 28.8 | 0.81 |
| 28 | 6 | 14.4490 | 38.2 | 2.56 |
| 31 | 6 | 11.7792 | 35.2 | 2.00 |
| Overall | 48 |  | 24.5 |  |

Test

| Null hypothesis | H₀: All medians are equal |
| --- | --- |
| Alternative hypothesis | H₁: At least one median is different |

| DF | H-Value | P-Value |
| --- | --- | --- |
| 7 | 17.97 | 0.012 |

Kruskal-Wallis Test: Nitrification vs Date

Ammonium

Descriptive Statistics

| Day | N | Median | Mean Rank | Z-Value |
| --- | --- | --- | --- | --- |
| 3 | 6 | 151.045 | 35.8 | 2.12 |
| 4 | 6 | 146.325 | 30.8 | 1.18 |
| 5 | 6 | 140.502 | 19.2 | -1.00 |
| 6 | 6 | 141.432 | 22.5 | -0.37 |
| 7 | 6 | 141.241 | 22.8 | -0.31 |
| 8 | 6 | 137.218 | 18.8 | -1.06 |
| 9 | 6 | 141.107 | 24.5 | 0.00 |
| 10 | 6 | 138.755 | 21.5 | -0.56 |
| Overall | 48 |  | 24.5 |  |

Test

| Null hypothesis | H₀: All medians are equal |
| --- | --- |
| Alternative hypothesis | H₁: At least one median is different |

| DF | H-Value | P-Value |
| --- | --- | --- |
| 7 | 7.50 | 0.379 |

Kruskal-Wallis Sulphate reduction vs Date

Ammonium

Descriptive Statistics

| Day | N | Median | Mean Rank | Z-Value |
| --- | --- | --- | --- | --- |
| 3 | 6 | 100.396 | 28.8 | 0.81 |
| 4 | 6 | 99.594 | 27.3 | 0.53 |
| 5 | 6 | 99.104 | 27.0 | 0.47 |
| 6 | 6 | 98.463 | 31.5 | 1.31 |
| 7 | 6 | 94.348 | 26.2 | 0.31 |
| 8 | 6 | 91.959 | 21.2 | -0.62 |
| 9 | 6 | 92.436 | 19.5 | -0.94 |
| 10 | 6 | 88.603 | 14.5 | -1.87 |
| Overall | 48 |  | 24.5 |  |

Test

| Null hypothesis | H₀: All medians are equal |
| --- | --- |
| Alternative hypothesis | H₁: At least one median is different |

| DF | H-Value | P-Value |
| --- | --- | --- |
| 7 | 6.76 | 0.454 |

Kruskal-Wallis Test: Denitrification vs Date

Ammonium

Descriptive Statistics

| Day | N | Median | Mean Rank | Z-Value |
| --- | --- | --- | --- | --- |
| 3 | 6 | 23.7491 | 36.8 | 2.31 |
| 4 | 6 | 22.4074 | 32.5 | 1.50 |
| 5 | 6 | 20.8725 | 31.2 | 1.25 |
| 6 | 6 | 19.5084 | 24.3 | -0.03 |
| 7 | 6 | 19.2762 | 21.5 | -0.56 |
| 8 | 6 | 19.5401 | 19.3 | -0.97 |
| 9 | 6 | 17.1704 | 12.8 | -2.18 |
| 10 | 6 | 18.0455 | 17.5 | -1.31 |
| Overall | 48 |  | 24.5 |  |

Test

| Null hypothesis | H₀: All medians are equal |
| --- | --- |
| Alternative hypothesis | H₁: At least one median is different |

| DF | H-Value | P-Value |
| --- | --- | --- |
| 7 | 14.74 | 0.040 |

Kruskal-Wallis Test: DNRA vs Date

Ammonium

Descriptive Statistics

| Day | N | Median | Mean Rank | Z-Value |
| --- | --- | --- | --- | --- |
| 3 | 6 | 10.2406 | 27.8 | 0.62 |
| 4 | 6 | 12.1757 | 30.7 | 1.15 |
| 5 | 6 | 9.0124 | 31.5 | 1.31 |
| 6 | 6 | 10.4176 | 28.3 | 0.72 |
| 7 | 6 | 7.5475 | 25.8 | 0.25 |
| 8 | 6 | 5.8964 | 18.3 | -1.15 |
| 9 | 6 | 6.4707 | 19.5 | -0.94 |
| 10 | 6 | 5.3185 | 14.0 | -1.96 |
| Overall | 48 |  | 24.5 |  |

Test

| Null hypothesis | H₀: All medians are equal |
| --- | --- |
| Alternative hypothesis | H₁: At least one median is different |

| DF | H-Value | P-Value |
| --- | --- | --- |
| 7 | 8.81 | 0.266 |

Kruskal-Wallis Test: Nitrification vs Date

Nitrate

Descriptive Statistics

| Day | N | Median | Mean Rank | Z-Value |
| --- | --- | --- | --- | --- |
| 3 | 6 | 139.832 | 41.0 | 3.09 |
| 4 | 6 | 142.684 | 43.2 | 3.49 |
| 5 | 6 | 125.994 | 34.3 | 1.84 |
| 6 | 6 | 68.548 | 24.7 | 0.03 |
| 7 | 6 | 37.420 | 10.8 | -2.56 |
| 8 | 6 | 42.146 | 11.2 | -2.49 |
| 9 | 6 | 41.465 | 9.7 | -2.77 |
| 10 | 6 | 64.497 | 21.2 | -0.62 |
| Overall | 48 |  | 24.5 |  |

Test

| Null hypothesis | H₀: All medians are equal |
| --- | --- |
| Alternative hypothesis | H₁: At least one median is different |

| DF | H-Value | P-Value |
| --- | --- | --- |
| 7 | 40.20 | 0.000 |

|  |  |  |
| --- | --- | --- |

Kruskal-Wallis Test: Sulphate reduction vs Date

Nitrate

Descriptive Statistics

| DayNum | N | Median | Mean Rank | Z-Value |
| --- | --- | --- | --- | --- |
| 7 | 6 | 97.6400 | 41.5 | 3.18 |
| 10 | 6 | 96.3107 | 39.0 | 2.71 |
| 14 | 6 | 84.1955 | 30.5 | 1.12 |
| 17 | 6 | 62.0858 | 14.3 | -1.90 |
| 21 | 6 | 49.9397 | 8.7 | -2.96 |
| 24 | 6 | 59.5485 | 12.0 | -2.34 |
| 28 | 6 | 65.6515 | 16.7 | -1.47 |
| 31 | 6 | 89.2332 | 33.3 | 1.65 |
| Overall | 48 |  | 24.5 |  |

Test

| Null hypothesis | H₀: All medians are equal |
| --- | --- |
| Alternative hypothesis | H₁: At least one median is different |

| DF | H-Value | P-Value |
| --- | --- | --- |
| 7 | 36.27 | 0.000 |

Kruskal-Wallis Test: Denitrification vs Date

Nitrate

Descriptive Statistics

| DayNum | N | Median | Mean Rank | Z-Value |
| --- | --- | --- | --- | --- |
| 7 | 6 | 24.5028 | 41.7 | 3.21 |
| 10 | 6 | 20.7683 | 36.7 | 2.28 |
| 14 | 6 | 17.8116 | 29.8 | 1.00 |
| 17 | 6 | 9.9186 | 15.2 | -1.75 |
| 21 | 6 | 7.5945 | 6.7 | -3.34 |
| 24 | 6 | 10.3818 | 14.2 | -1.93 |
| 28 | 6 | 11.3877 | 19.0 | -1.03 |
| 31 | 6 | 18.4539 | 32.8 | 1.56 |
| Overall | 48 |  | 24.5 |  |

Test

| Null hypothesis | H₀: All medians are equal |
| --- | --- |
| Alternative hypothesis | H₁: At least one median is different |

| DF | H-Value | P-Value |
| --- | --- | --- |
| 7 | 33.15 | 0.000 |

Kruskal-Wallis Test: DNRA vs Date

Nitrate

Descriptive Statistics

| DayNum | N | Median | Mean Rank | Z-Value |
| --- | --- | --- | --- | --- |
| 7 | 6 | 7.1342 | 16.7 | -1.47 |
| 10 | 6 | 6.2919 | 10.3 | -2.65 |
| 14 | 6 | 6.0051 | 8.2 | -3.06 |
| 17 | 6 | 10.8152 | 24.2 | -0.06 |
| 21 | 6 | 21.4184 | 33.3 | 1.65 |
| 24 | 6 | 17.6009 | 30.3 | 1.09 |
| 28 | 6 | 22.8048 | 33.3 | 1.65 |
| 31 | 6 | 25.2330 | 39.7 | 2.84 |
| Overall | 48 |  | 24.5 |  |

Test

| Null hypothesis | H₀: All medians are equal |
| --- | --- |
| Alternative hypothesis | H₁: At least one median is different |

| DF | H-Value | P-Value |
| --- | --- | --- |
| 7 | 29.05 | 0.000 |
