# Supplementary material for: Delayed Shift in Microbiota Composition in a Marine Microcosm Pollution Experiment
Source: Curr Microbiol. 2024 Sep 18;81(11):365. doi: 10.1007/s00284-024-03869-5 (PMC11410848; doi:10.1007/s00284-024-03869-5)
Supplement: Supplementary file 3 — Supplementary file3 (DOCX 31 KB) [file 284_2024_3869_MOESM3_ESM.docx]

**Supplementary Table 1 qPCR quantification of the 16S rRNA gene, species richness and distance decay information**

| **Condition** | **Selection** | **Incubation time (Days)** | **Gene copes per gram** | **Species riches** | **Distance decay** |
| --- | --- | --- | --- | --- | --- |
| oxic | ammonium | 7 | 7.08 | 4102 | 45.39 |
| oxic | ammonium | 7 | 7.62 | 3470 | 42.74 |
| oxic | ammonium | 7 | 7.79 | 4248 | 41.79 |
| anoxic | ammonium | 7 | 7.09 | 4416 | 46.34 |
| anoxic | ammonium | 7 | 8.15 | 3859 | 47.82 |
| anoxic | ammonium | 7 | 8.03 | 3866 | 40.06 |
| oxic | ammonium | 10 | 8.33 | 3458 | 49.86 |
| oxic | ammonium | 10 | 8.30 | 2663 | 60.15 |
| oxic | ammonium | 10 | 7.94 | 3912 | 76.12 |
| anoxic | ammonium | 10 | 7.70 | 2897 | 47.72 |
| anoxic | ammonium | 10 | 7.57 | 3405 | 54.65 |
| anoxic | ammonium | 10 | 8.04 | 3356 | 49.92 |
| oxic | ammonium | 14 | 8.10 | 3553 | 108.04 |
| oxic | ammonium | 14 | 8.26 | 3624 | 60.66 |
| oxic | ammonium | 14 | 8.09 | 4544 | 54.65 |
| anoxic | ammonium | 14 | 8.02 | 3641 | 56.95 |
| anoxic | ammonium | 14 | 7.91 | 3911 | 58.91 |
| anoxic | ammonium | 14 | 7.85 | 4622 | 54.39 |
| oxic | ammonium | 17 | 7.74 | 2337 | 53.16 |
| oxic | ammonium | 17 | 7.67 | 2743 | 54.76 |
| oxic | ammonium | 17 | 7.67 | 1695 | 56.49 |
| anoxic | ammonium | 17 | 7.63 | 1567 | 59.29 |
| anoxic | ammonium | 17 | 6.84 | 1558 | 51.39 |
| anoxic | ammonium | 17 | 7.43 | 1945 | 58.30 |
| oxic | ammonium | 21 | 6.71 | 2524 | 54.14 |
| oxic | ammonium | 21 | 7.44 | 2508 | 51.89 |
| oxic | ammonium | 21 | 7.45 | 2261 | 54.59 |
| anoxic | ammonium | 21 | 7.85 | 2370 | 62.49 |
| anoxic | ammonium | 21 | 8.05 | 3231 | 70.32 |
| anoxic | ammonium | 21 | 7.85 | 3469 | 60.43 |
| oxic | ammonium | 24 | 7.76 | 2861 | 59.50 |
| oxic | ammonium | 24 | 7.79 | 2708 | 55.54 |
| oxic | ammonium | 24 | 7.60 | 2456 | 54.79 |
| anoxic | ammonium | 24 | 7.92 | 2988 | 60.55 |
| anoxic | ammonium | 24 | 7.75 | 2382 | 55.64 |
| anoxic | ammonium | 24 | 8.12 | 3720 | 58.97 |
| oxic | ammonium | 28 | 8.10 | 3252 | 58.59 |
| oxic | ammonium | 28 | 8.08 | 2722 | 59.08 |
| oxic | ammonium | 28 | 7.95 | 2952 | 56.33 |
| anoxic | ammonium | 28 | 8.07 | 2931 | 61.72 |
| anoxic | ammonium | 28 | 7.68 | 2607 | 65.92 |
| anoxic | ammonium | 28 | 8.09 | 3165 | 64.72 |
| oxic | ammonium | 31 | 7.87 | 3719 | 57.76 |
| oxic | ammonium | 31 | 7.82 | 3646 | 56.24 |
| oxic | ammonium | 31 | 7.72 | 2981 | 58.19 |
| anoxic | ammonium | 31 | 7.98 | 3282 | 63.38 |
| anoxic | ammonium | 31 | 7.87 | 2863 | 60.19 |
| anoxic | ammonium | 31 | 7.95 | 3601 | 54.12 |
| oxic | nitrate | 7 | 7.98 | 2274 | 42.29 |
| oxic | nitrate | 7 | 7.47 | 3550 | 32.27 |
| oxic | nitrate | 7 | 7.88 | 3153 | 56.61 |
| anoxic | nitrate | 7 | 7.84 | 4579 | 38.15 |
| anoxic | nitrate | 7 | 7.55 | 2463 | 44.32 |
| anoxic | nitrate | 7 | 7.72 | 3715 | 74.28 |
| oxic | nitrate | 10 | 8.03 | 3150 | 80.32 |
| oxic | nitrate | 10 | 8.14 | 2806 | 80.51 |
| oxic | nitrate | 10 | 7.98 | 3712 | 79.21 |
| anoxic | nitrate | 10 | 7.99 | 3445 | 57.94 |
| anoxic | nitrate | 10 | 7.84 | 3949 | 95.69 |
| anoxic | nitrate | 10 | 7.63 | 3973 | 83.51 |
| oxic | nitrate | 14 | 7.71 | 3127 | 89.55 |
| oxic | nitrate | 14 | 7.94 | 3201 | 112.10 |
| oxic | nitrate | 14 | 7.78 | 1952 | 107.16 |
| anoxic | nitrate | 14 | 7.65 | 3987 | 100.64 |
| anoxic | nitrate | 14 | 7.82 | 3354 | 73.68 |
| anoxic | nitrate | 14 | 7.71 | 3496 | 81.57 |
| oxic | nitrate | 17 | 7.45 | 1497 | 307.41 |
| oxic | nitrate | 17 | 7.55 | 2295 | 131.40 |
| oxic | nitrate | 17 | 7.83 | 2408 | 192.97 |
| anoxic | nitrate | 17 | 7.59 | 1386 | 315.44 |
| anoxic | nitrate | 17 | 7.50 | 1589 | 269.75 |
| anoxic | nitrate | 17 | 7.83 | 1937 | 330.63 |
| oxic | nitrate | 21 | 8.08 | 1629 | 433.80 |
| oxic | nitrate | 21 | 7.84 | 1389 | 486.49 |
| oxic | nitrate | 21 | 7.78 | 1223 | 380.02 |
| anoxic | nitrate | 21 | 8.30 | 880 | 273.86 |
| anoxic | nitrate | 21 | 8.15 | 1074 | 476.39 |
| anoxic | nitrate | 21 | 8.11 | 986 | 327.64 |
| oxic | nitrate | 24 | 8.30 | 1643 | 460.65 |
| oxic | nitrate | 24 | 8.48 | 1675 | 466.91 |
| oxic | nitrate | 24 | 8.53 | 1717 | 460.09 |
| anoxic | nitrate | 24 | 8.58 | 1673 | 338.27 |
| anoxic | nitrate | 24 | 8.64 | 1378 | 348.82 |
| anoxic | nitrate | 24 | 8.67 | 1255 | 396.79 |
| oxic | nitrate | 28 | 8.68 | 1684 | 391.95 |
| oxic | nitrate | 28 | 8.46 | 1143 | 426.49 |
| oxic | nitrate | 28 | 8.71 | 1281 | 439.58 |
| anoxic | nitrate | 28 | 8.51 | 1560 | 530.43 |
| anoxic | nitrate | 28 | 8.57 | 1405 | 446.04 |
| anoxic | nitrate | 28 | 8.27 | 1216 | 378.13 |
| oxic | nitrate | 31 | 8.41 | 1300 | 302.06 |
| oxic | nitrate | 31 | 8.43 | 1922 | 273.65 |
| oxic | nitrate | 31 | 8.32 | 1612 | 341.81 |
| anoxic | nitrate | 31 | 8.10 | 1594 | 382.37 |
| anoxic | nitrate | 31 | 8.40 | 1306 | 341.42 |
| anoxic | nitrate | 31 | 8.44 | 1318 | 376.02 |
| oxic | sulfate | 7 | 7.35 | 3426 | 34.27 |
| oxic | sulfate | 7 | 7.88 | 3800 | 46.62 |
| oxic | sulfate | 7 | 8.08 | 4036 | 41.95 |
| anoxic | sulfate | 7 | 7.69 | 3657 | 40.91 |
| anoxic | sulfate | 7 | 7.89 | 3309 | 47.71 |
| anoxic | sulfate | 7 | 7.78 | 4680 | 42.06 |
| oxic | sulfate | 10 | 7.70 | 2866 | 90.87 |
| oxic | sulfate | 10 | 7.76 | 3759 | 46.51 |
| oxic | sulfate | 10 | 8.09 | 2330 | 53.18 |
| anoxic | sulfate | 10 | 7.83 | 3519 | 72.96 |
| anoxic | sulfate | 10 | 8.35 | 3276 | 87.11 |
| anoxic | sulfate | 10 | 8.22 | 3864 | 58.58 |
| oxic | sulfate | 14 | 7.35 | 1760 | 94.05 |
| oxic | sulfate | 14 | 7.48 | 1433 | 84.10 |
| oxic | sulfate | 14 | 7.55 | 1684 | 114.92 |
| anoxic | sulfate | 14 | 7.34 | 1997 | 59.66 |
| anoxic | sulfate | 14 | 2.70 | 2945 | 83.63 |
| anoxic | sulfate | 14 | 2.70 | 2672 | 110.11 |
| oxic | sulfate | 17 | 7.34 | 839 | 182.71 |
| oxic | sulfate | 17 | 7.54 | 1926 | 68.54 |
| oxic | sulfate | 17 | 7.32 | 956 | 78.44 |
| anoxic | sulfate | 17 | 7.41 | 2006 | 272.62 |
| anoxic | sulfate | 17 | 7.51 | 1971 | 303.41 |
| anoxic | sulfate | 17 | 7.15 | 1502 | 299.12 |
| oxic | sulfate | 21 | 7.30 | 2289 | 307.84 |
| oxic | sulfate | 21 | 7.86 | 2194 | 252.29 |
| oxic | sulfate | 21 | 7.65 | 1965 | 170.36 |
| anoxic | sulfate | 21 | 8.10 | 475 | 217.98 |
| anoxic | sulfate | 21 | 8.08 | 2464 | 164.02 |
| anoxic | sulfate | 21 | 7.79 | 2148 | 236.59 |
| oxic | sulfate | 24 | 8.03 | 3524 | 185.79 |
| oxic | sulfate | 24 | 7.85 | 2609 | 150.86 |
| oxic | sulfate | 24 | 8.02 | 3248 | 157.34 |
| anoxic | sulfate | 24 | 8.07 | 2581 | 214.16 |
| anoxic | sulfate | 24 | 7.20 | 2709 | 159.98 |
| anoxic | sulfate | 24 | 8.15 | 2033 | 332.97 |
| oxic | sulfate | 28 | 7.88 | 634 | 207.48 |
| oxic | sulfate | 28 | 8.02 | 3020 | 136.18 |
| oxic | sulfate | 28 | 7.89 | 2367 | 147.64 |
| anoxic | sulfate | 28 | 8.09 | 2234 | 168.24 |
| anoxic | sulfate | 28 | 7.93 | 1757 | 160.16 |
| anoxic | sulfate | 28 | 7.35 | 2684 | 203.05 |
| oxic | sulfate | 31 | 8.08 | 2457 | 142.12 |
| oxic | sulfate | 31 | 8.20 | 2944 | 118.77 |
| oxic | sulfate | 31 | 7.96 | 3230 | 84.59 |
| anoxic | sulfate | 31 | 8.31 | 2025 | 315.12 |
| anoxic | sulfate | 31 | 8.33 | 2312 | 205.49 |
| anoxic | sulfate | 31 | 7.88 | 2466 | 162.79 |
